# Mesenchymal stromal cells are more effective than the MSC secretome in diminishing injury and enhancing recovery following ventilator-induced lung injury

**DOI:** 10.1186/s40635-015-0065-y

**Published:** 2015-10-15

**Authors:** Mairead Hayes, Gerard F. Curley, Claire Masterson, James Devaney, Daniel O’Toole, John G. Laffey

**Affiliations:** Regenerative Medicine Institute, National University of Ireland, Galway, Ireland; Anaesthesia, School of Medicine, Clinical Sciences Institute, National University of Ireland, Galway, Ireland; Department of Anesthesia, Keenan Research Centre for Biomedical Science of St Michael’s Hospital, St. Michael’s Hospital, 30 Bond Street, Toronto, ON M5B 1W8 Canada; Department of Anesthesia, University of Toronto, Toronto, Canada

**Keywords:** Acute respiratory distress syndrome, Inflammation, Ventilation-induced lung injury, Repair, Mesenchymal stem/stromal cell

## Abstract

**Background:**

The potential for mesenchymal stem cells (MSCs) to reduce the severity of experimental lung injury has been established in several pre-clinical studies. We have recently demonstrated that MSCs, and MSC-secreted factors (secretome), enhance lung repair and regeneration at 48 h following ventilation-induced lung injury (VILI). We wished to determine the potential for MSC therapy to exert beneficial effects in the early recovery phase following VILI when ongoing injury coexists with processes of repair, and to compare the efficacy of MSC therapy to the use of the secretome alone.

**Methods:**

Male Sprague–Dawley rats were anesthetized, oro-tracheally intubated, and subjected to high stretch mechanical ventilation until lung compliance had declined by 50 % of baseline. Animals were then weaned from mechanical ventilation, and anesthesia discontinued. Once awake and spontaneously ventilating, animals received an intravenous injection of either rodent MSCs (10 million/kg), MSC-conditioned medium, fibroblasts (10 million/kg), or vehicle. Thereafter, the animals were allowed to recover and the extent of lung injury/repair was determined after 4 h.

**Results:**

Treatment with MSCs diminished injury and enhanced recovery following VILI to a greater extent compared to MSC-conditioned medium, with fibroblasts proving ineffective. MSCs, but not their conditioned medium, attenuated indices of lung injury including oxygenation, respiratory compliance, and lung edema. Total lung water as assessed by wet:dry ratio, bronchoalveolar lavage total inflammatory cell, neutrophil counts, and alveolar IL-6 concentrations were reduced in the animals that received MSC therapy.

**Conclusions:**

The immunomodulating and/or reparative effect of MSCs is evident early after VILI in this model. MSC-conditioned medium was not as effective as the cells themselves in diminishing injury and restoring lung structure and function.

## Background

The therapeutic potential of mesenchymal stem/stromal cells (MSCs) for acute respiratory distress syndrome (ARDS) has been demonstrated in several pre-clinical studies, in both sepsis-induced [1–4] and non-septic [5, 6] models of ARDS. However, many earlier studies in pre-clinical ARDS models are limited by the fact that MSCs were given at the time of, or even before, the induction of lung injury [1, 7]. This issue is of critical importance to the eventual therapeutic potential of MSCs, as in the clinical setting much of the injury process may be complete before the therapy can be given. A therapy that diminishes damaging inflammation and enhances injury resolution, applied after the injury has been induced, may therefore be more likely to translate successfully to the bedside and to be beneficial in the clinical setting. The demonstration that MSCs can enhance repair following severe ventilator-induced lung injury (VILI) offers considerable hope for the therapeutic potential of these cells [8, 9].

An important factor in identifying the optimal timing of administration for MSCs in ARDS is to establish the earliest time-point at which MSCs exert their beneficial effects after injury in the lung. While our group has established that MSCs exert therapeutic effects on lung repair when assessed 48 h after VILI, the timing of onset of benefit is unknown. Furthermore, the precise mechanism which underlies the beneficial effects of MSCs in ARDS remains to be determined. Several studies have reported that MSCs act via a paracrine mechanism, with soluble factors of the MSC secretome accounting for immunomodulatory and reparative effects [3, 4, 6, 10, 11]. In some studies, the beneficial effects of MSC administration have been replicated by the MSC ‘secretome’ alone [6, 8, 9, 12]. In contrast, other work suggests a central role for direct cell-to-cell contact by MSCs in mediating their beneficial effects [13]. The relative importance of the different mechanisms of action of MSCs may vary depending on the precise injury mechanism and phase of the disease process.

Because of these issues, we wished to determine the potential for MSC therapy to exert beneficial effects in the early recovery phase following VILI, and to compare the efficacy of MSC therapy to the use of the MSC secretome alone. We hypothesized that both MSCs and MSC-conditioned medium would diminish injury and enhance early recovery following ventilator-induced lung injury.

## Methods

All work was approved by the Animal Ethics Committee of the National University of Ireland, Galway and conducted under license from the Department of Health, Ireland. Specific-pathogen-free adult male Sprague–Dawley rats (Charles River Laboratories, Kent, UK) weighing between 400 and 500 g were used in all experiments.

### MSC and fibroblast culture and characterization

Rodent MSCs (MSCs) were isolated from rat femora and tibiae under sterile conditions as previously described [14]. Briefly, male Sprague–Dawley rats (8–12 weeks old) were euthanized by inhalation of CO_2_. Incisions were made on both lower limbs to expose the tibiae and femora. Both bones were removed from the hind limbs and placed in ice cold sterile Tyrode’s solution (Sigma, St. Louis, MO). The marrow was then flushed into a dish containing MSC complete culture medium (MEM-α Media (Gibco, Paisley, UK), F12-Ham Media (Gibco), 10 % fetal bovine serum (PAA, Somerset, UK), and 1 % antibiotic/antimycotic (Sigma) and dispersed into a cell suspension. After centrifugation and filtration through a 100-μm nylon mesh, a cell count was performed and the cells were transferred to a 175-cm^2^ flasks containing 30 ml of MSC complete medium, at a density of 9 × 10^5^ cells/cm^2^. On day 3 of culture in an atmosphere of 5 % CO_2_/90 % humidity at 37 °C, medium and non-adherent cells were removed and fresh medium was added to each flask. Cells were ready for subculture (usually after 16–17 days) when colonies began to exhibit a compact appearance and multi-layered growth or when the loosely formed colonies began to merge into a monolayer (<90 % of confluence).

Thereafter, cells were ready to be passaged after 6/7 days culture, at 80 % confluence. For passage, media was aspirated off and cells were washed with sterile PBS to remove any remaining serum. Eight milliliters 0.25 % trypsin/EDTA solution was added to the cells, which were incubated for 5 min at 37 °C. The enzymatic reaction was stopped by adding the same volume of MSC media to cells. Cells were centrifuged at 400×*g* for 5 min. Media was aspirated off the cell pellet which was resuspended in 1 ml and a hemocytometer count was undertaken. Cells were expanded to passage 4, whereupon they were used for experiments. MSC phenotype was confirmed via characterization of cell surface markers [antibodies to CD31, CD34, CD44, CD45, CD54, CD73, and CD90, Santa Cruz Biotechnology, Santa Cruz, CA] and analyzed with a FACScan (Becton Dickinson, Franklin Lakes, NJ) and CellQuest software as described [15, 16].

Fibroblasts, used as control cells, were obtained from the dermis of adult Sprague–Dawley rats as previously described [9]. Following euthanization, the skin and subcutaneous tissue strips were excised from the ventral surface of the rat abdominal wall, placed into 70 % ethanol for 30 s, and then placed in 0.25 % trypsin (Sigma) overnight. The epidermis was then peeled from the dermal layer, and the dermal layer was placed on a scored six-well plate (Sarstedt, Wexford, Ireland) in F-12/MEM-α medium supplemented with fetal calf serum (10 %) and penicillin/streptomycin (1 %).

### MSC-conditioned medium

MSC-conditioned medium was generated from allogeneic rat MSCs (4 × 10^6^). Briefly, the cells were washed, the medium was replaced, and the subsequent medium without serum for the next 24 h was used as the conditioned medium (CM). All conditioned medium was sterile filtered through a 22-μm filter to remove cellular debris and concentrated using a 3000 kDa centrifugal concentrating filter (Amicon, Billerica, MA, USA) to give 500 μl (i.e., concentrated ×30).

### Rodent ventilator-induced injury protocol

**Fig. 1:**
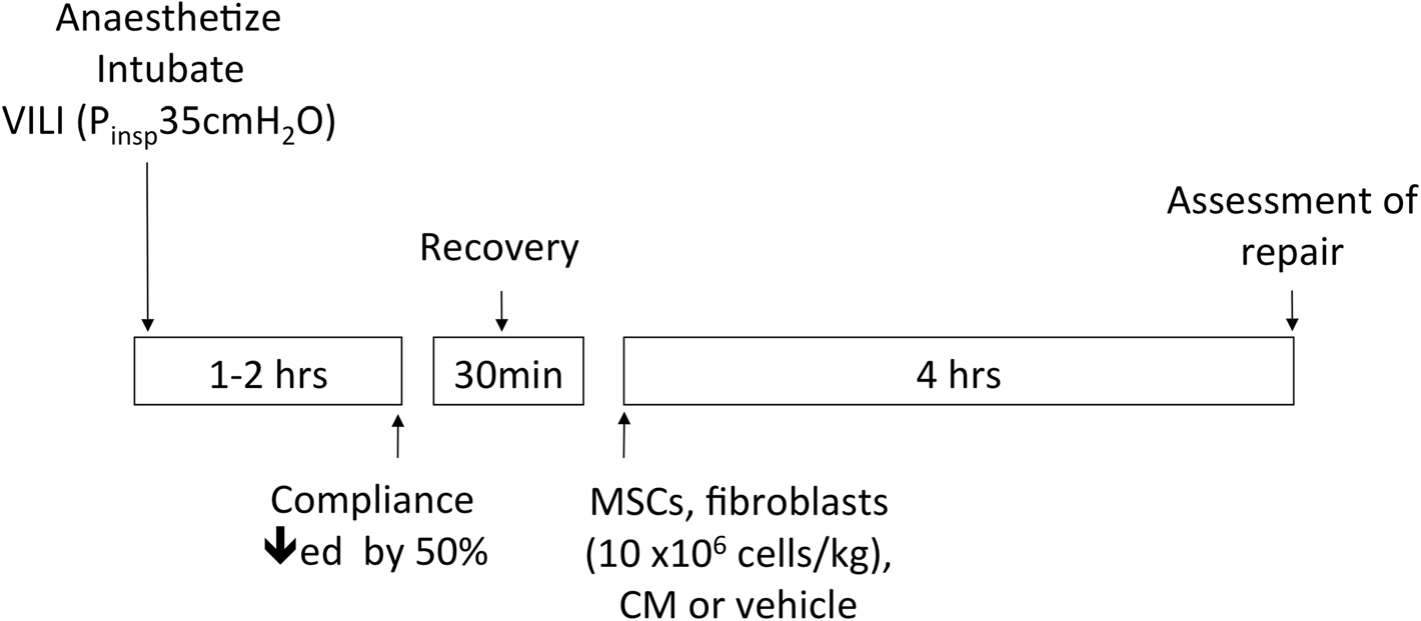
Flow diagram indicating timelines for experimental interventions

We utilized our established model of repair from VILI as previously described (Fig. 1) [8, 9, 17]. Briefly, anesthesia was induced with intraperitoneal ketamine 80 mg kg^−1^ (Ketalar, Pfizer, Cork, Ireland) and xylazine 8 mg kg^−1^ (Xylapan, Vétoquinol, Dublin, Ireland). After confirmation of depth of anesthesia by paw clamp, intravenous access was obtained via tail vein, laryngoscopy was performed and the animals were intubated with a size 14G intravenous catheter (BD Insyte®, Becton Dickinson Ltd, Oxford, UK). The lungs were ventilated using a small animal ventilator (CWE SAR 830 AP, CWE Inc, Pennsylvania, USA). Anesthesia was maintained with repeated boli of Saffan® (alfaxadone 0.9 % and alfadadolone acetate 0.3 %; Schering Plough, Welwyn Garden City, UK) and muscle relaxation was achieved with cis-atracurium besylate 0.5 mg kg^−1^ (GlaxoSmithKline, Dublin, Ireland). Animals were subjected to injurious mechanical ventilation, using the following ventilator settings: P_insp_ 35 cmH_2_O, respiratory rate 18 min^−1^, and PEEP 0 cmH_2_O until compliance decreased by 50 % of baseline. Compliance was determined by incremental syringe injection of 1 ml aliquots of air over 1 s, while simultaneously recording plateau airway pressure after each injection. Final compliance was calculated by determining plateau airway pressure after 5 ml volume injection. At this point, high stretch ventilation was discontinued and animals were extubated, allowed to regain consciousness, and entered into the treatment protocol. Following recovery, animals were randomized to intravenous administration of: (i) 10 × 10^6^/kg allogeneic rat MSCs (MSC group), (ii) 500 μl phosphate buffered saline (vehicle group), (iii) 10 × 10^6^/kg rat dermal fibroblasts (fibroblast group), or (iv) 500 μl of conditioned medium from 10 × 10^6^/kg allogeneic rat MSCs (MSC-CM group), in a four-group design.

### Assessment of injury and repair

Four hours following VILI induction, animals were re-anesthetized. A tracheostomy was inserted and carotid arterial access established (22G, BD Insyte), and the lungs were mechanically ventilated at a respiratory rate of 80 min^−1^, tidal volume 6 ml kg^−1^, and positive end-expiratory pressure 2 cmH_2_O as previously described [18–20]. Intra-arterial blood pressure, peak airway pressures, and rectal temperature were recorded continuously. Static inflation lung compliance measurements were performed as previously described [18, 21]. After 20 min, the inspired gas was altered to FiO_2_ of 1.0 for 15 min, and a final arterial blood sample was taken. Heparin (400 IU kg^−1^, CP Pharmaceuticals, Wrexham, U.K.) was then administered intravenously, and animals were killed by exsanguination.

Immediately post-mortem, the heart–lung block was dissected and bronchoalveolar lavage (BAL) collection was performed as previously described [20, 22]. BAL differential cell counts were performed. Protein concentration was determined using a Micro BCA™ Protein assay kit (Pierce, Rockford, IL, USA) [23]. BAL IL-1β, IL-6, IL-10, and KGF concentrations were determined using quantitative sandwich enzyme-linked immunosorbent assays (R and D Systems, Abingdon, UK) as previously described [18–20].

Lung wet:dry weight ratios were determined using the lowest lobe of the right lung as previously described [24]. The left lung was isolated and fixed for morphometric examination. Briefly, following perfusion of the pulmonary circulation with heparinized saline, paraformaldehyde was then instilled through the pulmonary artery catheter at a pressure of 62.5 cmH_2_O, and the left lung inflated with paraformaldehyde (4 % wt vol^−1^) in phosphate buffered saline (300 mOsmol) at a pressure of 25 cmH_2_O via the tracheal catheter for 30 min. The pulmonary artery and trachea were then ligated, and the lung was stored in paraformaldehyde. The extent of histologic lung damage was determined using quantitative stereological techniques as previously described [22, 24]. Briefly, the vertical axis of each left lung was identified and the lung was cut perpendicular to this axis into 4-mm-thick slices, which were then embedded in paraffin and sections (7 μm) cut from each slice, mounted on slides, and stained with hematoxylin and eosin. An image of each complete lung section was captured, a point counting grid was superimposed on the image, and the proportions of intra-acinar airspace and tissue determined in 3–5 randomly chosen visual fields sampled from each section under light microscopy (×10 objective; Leica, Laboratory instruments, Wetzlar, Germany). Intra-acinar tissue was defined as all tissues within the gas exchange portion of the lung, i.e., respiratory bronchioles, alveolar ducts, alveolar sacs, and alveoli, including blood vessels contained within their walls. The intra-acinar airspace was defined as all airspaces within the lumen of respiratory bronchioles, alveolar ducts, alveolar sacs, and alveoli.

### Statistical analysis

The distribution of all data was tested for normality using Kolmogorov-Smirnov tests. Data were analyzed by one-way ANOVA, followed by Student-Newman-Keuls, or by Kruskal-Wallis followed by Mann–Whitney *U* test with the Bonferroni correction for multiple comparisons, as appropriate. Underlying model assumptions were deemed appropriate on the basis of suitable residual plots. A two-tailed *p* value of <0.05 was considered significant.

## Results

Thirty-six animals were entered into the experimental protocol, with 9 allocated to each of the 4 groups. Animals were randomly allocated into treatment groups prior to anesthesia and VILI, by means of sealed envelopes containing group assignments. One investigator, who was not involved in the animal experiments, was responsible for group assignment and preparation of the cells and conditioned medium for administration. A separate investigator was responsible for physiologic measurements, as well as estimates of inflammatory cell infiltration, histology, and cytokine ELISAs. This investigator was blinded to group allocation. Three animals, one each allocated to receive MSCs, fibroblasts, and MSC-CM group did not survive the induction of VILI and so did not receive their allocated treatment. All other animals survived the injury protocol and subsequent treatment allocation. There were no further deaths in the 4-h period between receiving the treatment and the end of the protocol. There were no differences among the groups at baseline in terms of pre-injury variables, the duration of injurious ventilation or the extent of the lung injury produced (Table 1).

**Table 1:** Baseline data regarding the four groups

Variable	Vehicle^a^	Mesenchymal stem cell	Conditioned medium	Fibroblast therapy
Number of animals injured	9	9	9	9
Number of animals treated	9/9	8/9	8/9	8/9
Animal survival post treatment (%)	9 (100 %)	8 (100 %)	8 (100 %)	8 (100 %)
Animal weight (g)	448 ± 19	476 ± 35	509 ± 25	450 ± 12
Duration of injurious ventilation (min)	108 ± 39	120 ± 40	131 ± 66	101 ± 59
Lung compliance pre-injury (ml/mmHg)	0.78 ± 0.14	0.75 ± 0.1	0.79 ± 0.14	0.74 ± 0.05
Lung compliance immediately post-VILI	0.37 ± 0.5	0.4 ± 0.04	0.41 ± 0.05	0.37 ± 0.03
Change in compliance from baseline (%)	50 ± 7	49 ± 4	48 ± 5	49 ± 5

Data are expressed as mean ± SD

*VILI* ventilation-induced lung injury

^a^Treatment with vehicle alone

### MSCs restored lung function following VILI

**Fig. 2:**
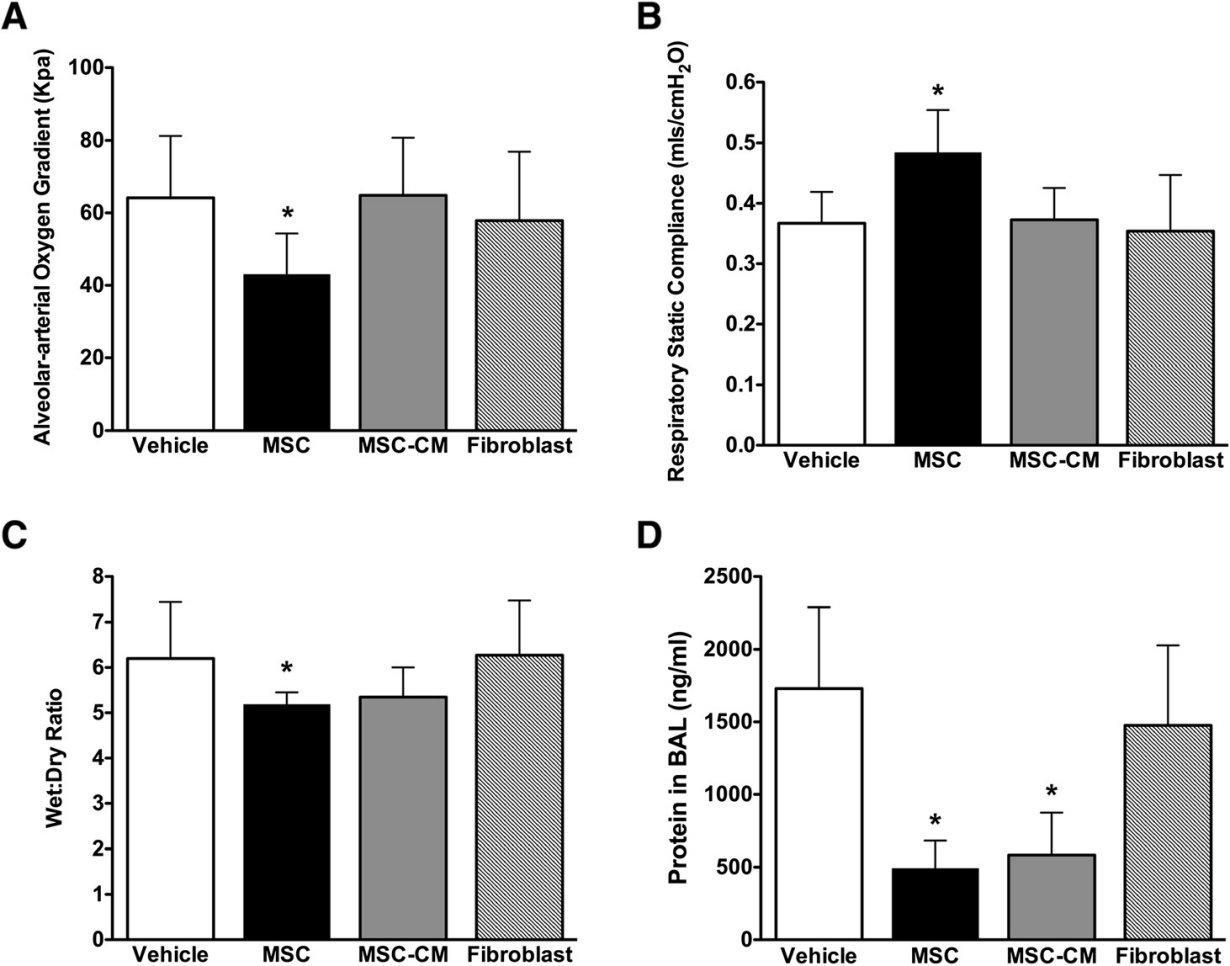
Mesenchymal stem cells attenuate physiological lung injury 4 h after VILI. Histograms representing alveolar–arterial oxygen gradient (**a**), static lung compliance (**b**), lung wet:dry weight (**c**), and BAL protein (**d**) 4 h following treatment. Abbreviations: *Vehicle* animals that received intravenous phosphate buffered saline alone, *MSC* mesenchymal stem cells, *MSC-CM* MSC-conditioned medium, *BAL* bronchoalveolar lavage. *Significantly different from vehicle control groups (*P* <0.05, ANOVA)

Intravenous MSC therapy enhanced restoration of arterial oxygenation, as evidenced by a reduced alveolar–arterial oxygen gradient compared to vehicle and fibroblast therapy (Fig. 2a). MSC therapy restored respiratory system static compliance to a greater degree than fibroblast or vehicle therapy (Fig. 2b). Compliance after 4 h remained unaltered in the vehicle and fibroblast groups (52 ± 11 % decrease from baseline and 52 ± 2 % decrease from baseline, respectively) versus partial restoration in the MSC group (34 ± 13 % decrease from baseline). Of interest, MSC-CM did not restore lung physiologic function, as measured by oxygenation (*p* = 0.94 vs vehicle group; *p* = 0.007 vs MSC group) or lung compliance (53 ± 5 % decrease from baseline, *p* = 0.83 vs vehicle; *p* = 0.003 vs MSC group) (Fig. 2a, b). MSC therapy decreased lung wet:dry weight ratios (Fig. 2c, *p* = 0.038 for MSC vs vehicle; *p* = 0.52 for MSC vs MSC-CM**)**, while both MSC and MSC-CM therapy decreased alveolar fluid protein concentrations (Fig. 2d, *p* <0.001 for MSC vs vehicle and MSC-CM vs vehicle; *p* = 0.46 for MSC vs MSC-CM).

### MSCs modulated the inflammatory response to VILI

**Fig. 3:**
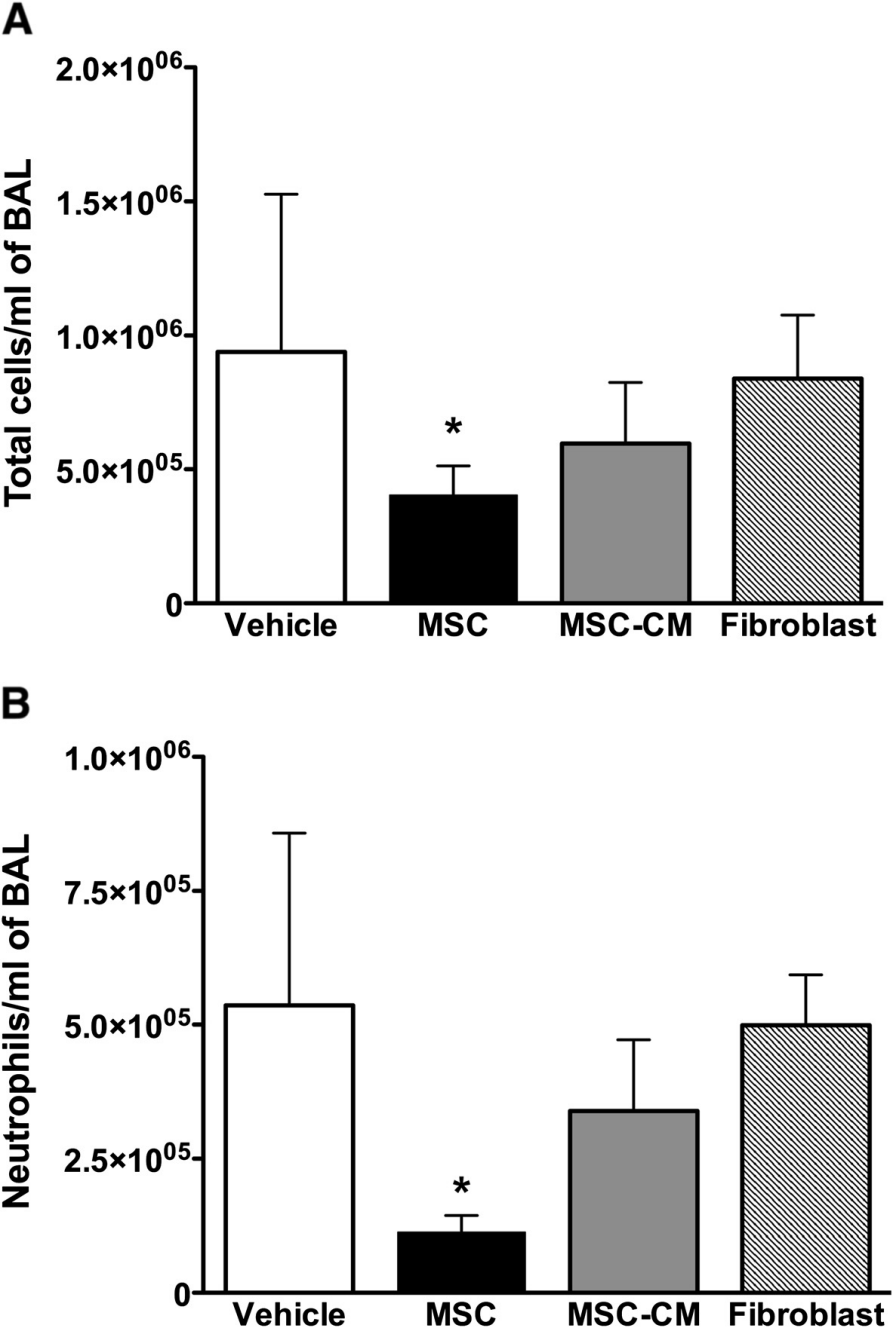
MSCs modulate inflammation after VILI. Histogram representing bronchoalveolar lavage total white cell count (**a**), and the proportion of neutrophils in the bronchoalveolar lavage (**b**) 4 h following treatment. Abbreviations: *Vehicle* animals that received intravenous phosphate buffered saline alone, *MSC* mesenchymal stem cells, *MSC-CM* MSC-conditioned medium, *BAL* bronchoalveolar lavage. *Significantly different from vehicle control groups (*P* <0.05, ANOVA)

MSC therapy decreased total inflammatory cell counts in BAL fluid (Fig. 3a) and substantially attenuated lung neutrophil accumulation (*p* <0.001) (Fig. 3b). In contrast, there was no effect of MSC-CM on alveolar inflammatory cell infiltration (*p* = 0.14 vs control, *p* = 0.06 vs MSC group) or in the number of BAL neutrophils present compared to vehicle or fibroblast therapy (*p* = 0.128 vs vehicle, *p* <0.001 vs MSC group).

**Fig. 4:**
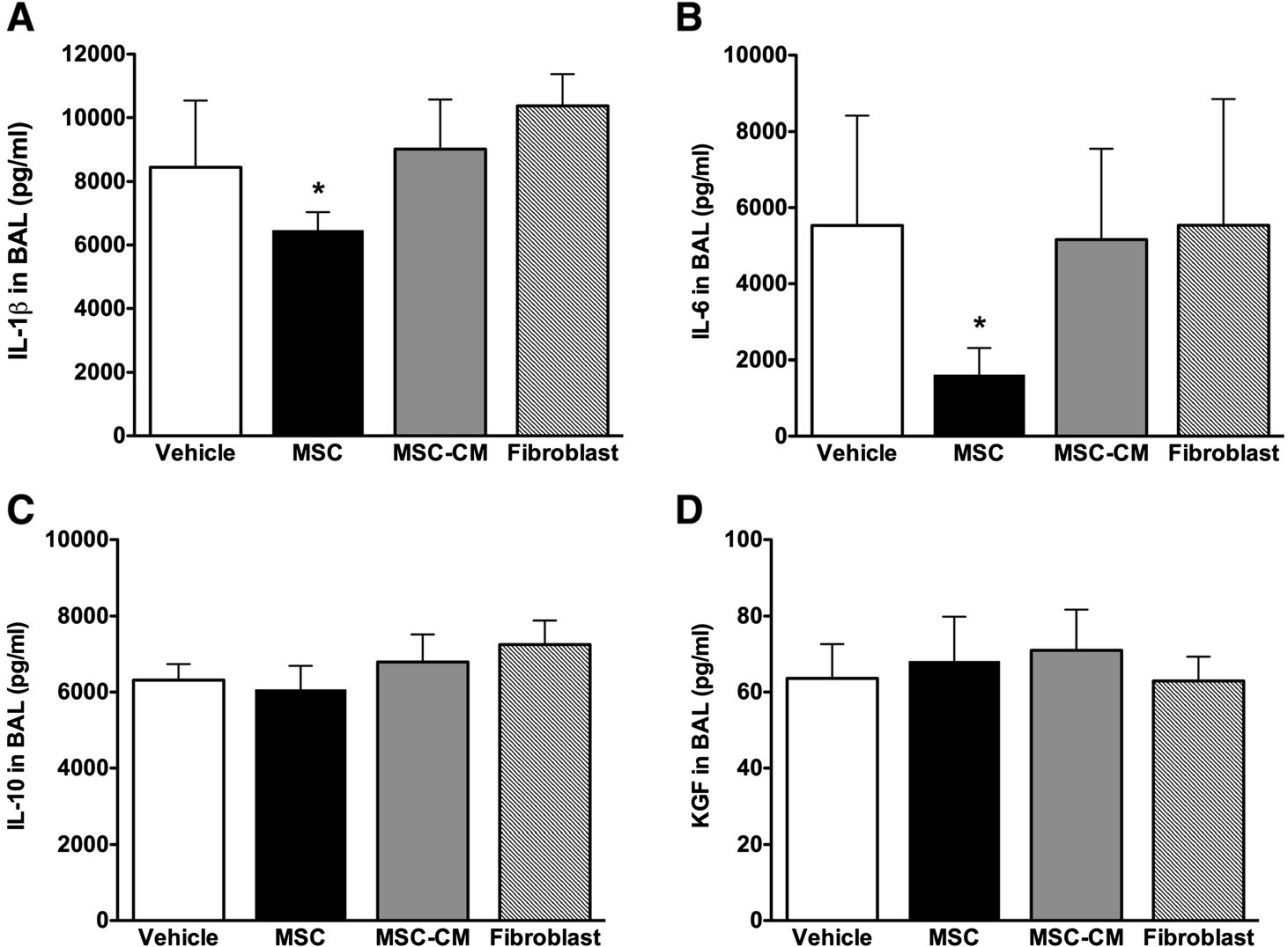
MSCs modulate cytokine expression 4 h after VILI. Histograms representing bronchoalveolar lavage IL-1β (**a**), IL-6 (**b**), IL-10 (**c**), and KGF (**d**) concentrations 4 h following treatment. Abbreviations: *Vehicle* animals that received intravenous phosphate buffered saline alone, *MSC* mesenchymal stem cells, *MSC-CM* MSC-conditioned medium, *BAL* bronchoalveolar lavage, *IL* interleukin, *KGF* keratinocyte growth factor. *Significantly different from vehicle control groups (*P* <0.05, ANOVA)

MSC therapy decreased alveolar concentrations of IL-1β and IL-6 (Fig. 4a, b). In contrast, there was no effect of MSC treatment on alveolar concentration of the anti-inflammatory cytokine IL-10 or keratinocyte growth factor (Fig. 4c, d). MSC-CM did not modulate the inflammatory response.

### MSCs restore lung structure following VILI

**Fig. 5:**
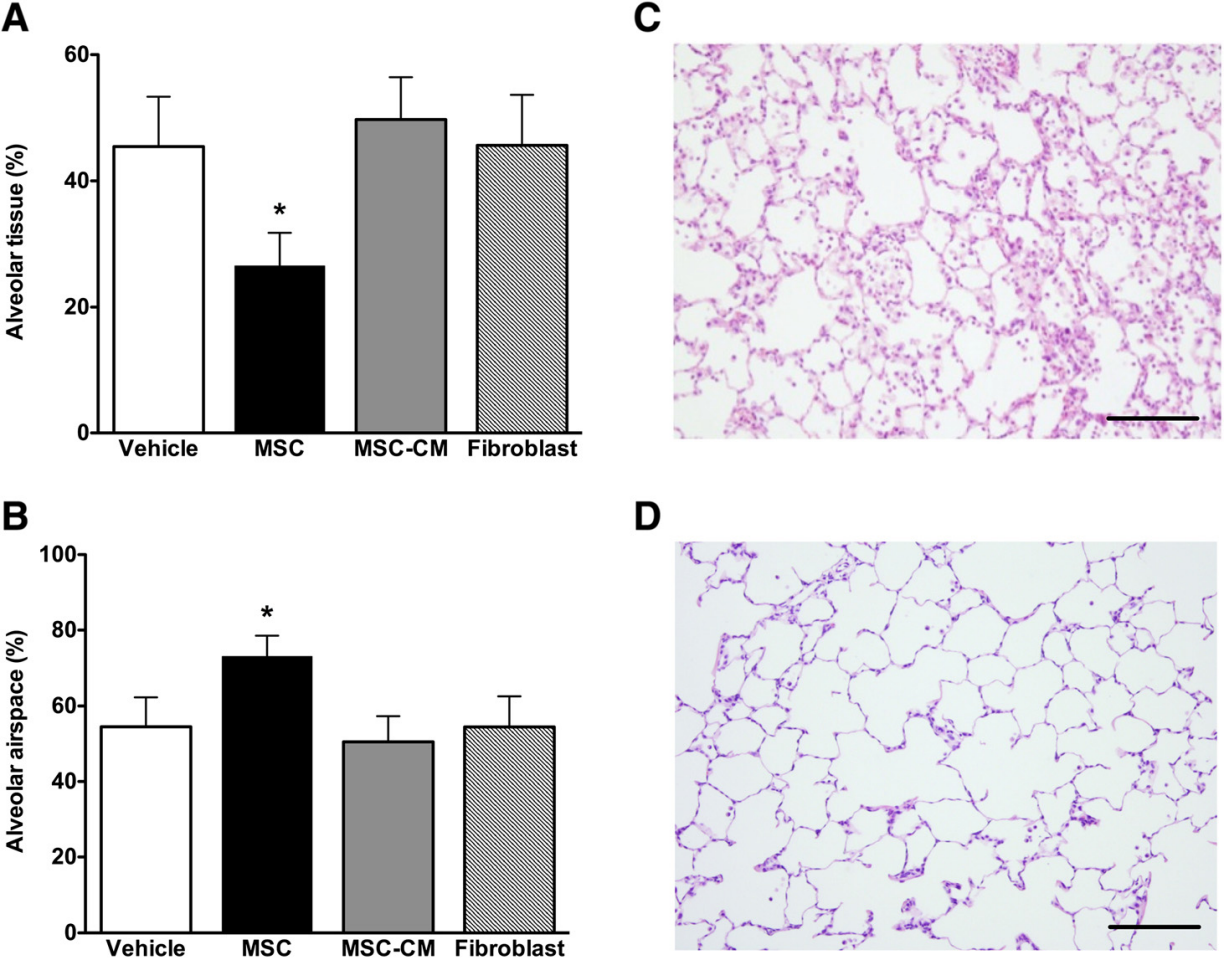
MSCs enhance resolution of structural lung injury 4 h after VILI. Histograms demonstrating enhanced resolution of structural lung injury evidenced by decreased alveolar lung tissue (**a**) and increased alveolar airspace fraction (**b**) in the MSC group. Representative photomicrographs of lung taken at a magnification of ×10 from a PBS control (**c**), and MSC-treated (**d**), animal demonstrate greater resolution of lung injury with MSCs at 4 h. The *scale bar* at the *bottom right* of each figure represents 200 μm

MSCs decreased alveolar thickening, as evidenced by reduced alveolar tissue volume fraction, and increased recovery of airspace volume, as evidenced by increased alveolar airspace volume fraction (Fig. 5a, b). Representative histological sections of lung demonstrate the greater degree of resolution of injury and alveolar infiltrates in the MSC-treated animals compared to the vehicle-treated animals (Fig. 5c, d). In contrast, there was no evidence of attenuation of structural lung damage in the conditioned medium group compared to vehicle or fibroblast treatment (Fig. 5).

## Discussion

This study was designed to examine the effects of intravenous MSCs and the MSC secretome on recovery of lung function and structure in the early phase following lung injury induced by high stretch mechanical ventilation. Given the recently described beneficial effects of MSCs in VILI, when administered both in the repair phase [8, 9] and as a pre-treatment to decrease VILI development [7], we postulated that MSCs would enhance recovery at an early time-point after VILI. We furthermore hypothesized that the MSC secretome would have similar effects to the cells themselves on recovery after VILI. We found that the MSC therapy enhanced resolution of lung injury but that the secretome alone was less effective at this stage in the recovery process.

### MSCs augment early recovery following VILI

MSC therapy enhanced resolution of the lung injury as early as 4 h after VILI. MSC-treated lungs had significantly enhanced function, as evidenced by improved oxygenation and lung compliance, and decreased lung oedema, compared to vehicle or fibroblast therapy. MSC therapy modulated the immune response to VILI, decreasing alveolar neutrophil infiltration, a major mediator of lung injury in VILI. MSC therapy decreased alveolar concentrations of the pro-inflammatory cytokines IL-1β and IL-6. IL-6 is among the most biologically active cytokines in the lung in VILI [25] and has been shown to contribute to neutrophil accumulation and alveolar barrier disruption [26]. Lower tidal volume ventilation, the only intervention shown to reduce mortality from ARDS, is associated with a decrease in IL-6 [27]. In the NIH/ARDSnet study, plasma levels of IL-6 in patients with ARDS were positively correlated with mortality [28]. In contrast to our earlier studies [8, 17] and to findings in other models [1], we found no effect of MSCs on alveolar IL-10 concentrations. However, increased production of IL-10 is not universally observed with MSC therapy [2] and may depend on the injury mechanism and the stage of evolution of the injury and repair processes. For example, Danchuk et al. found no significant rise in pulmonary IL-10 in MSC-treated mice 24 and 48 h after LPS injury [29].

### MSCs versus the MSC secretome for repair of the lung following VILI

In these experiments, the MSC secretome was largely ineffective in restoring lung function or structure or modulating the immune response, in the early stages following VILI. This contrasts with the prior demonstration from our group that the MSC secretome was as effective as MSC therapy itself in repairing the lung at 48 h following VILI [8, 9].

The original concept that MSCs could induce tissue regeneration by differentiation into the cells of the injured organ has been largely disproven [30]. Multiple individual paracrine factors in the MSC secretome have been demonstrated to play important roles in mediating the effects of MSCs [12], highlighting the diverse mechanisms of action of these cells. In our 48-h model of recovery from VILI, MSCs and their conditioned medium enhanced physiologic recovery and diminished indices of inflammation [17]. Our in vitro wound repair studies [17], and studies of alveolar fluid clearance in the isolated human lung [31, 32], indicate an important role for MSC-secreted factors, particularly Keratinocyte Growth Factor. The fact that MSCs are present only transiently at the site of injury in animal models further supports this hypothesis [8]. These findings raise the possibility that mediators released by cells could be isolated and delivered systemically or locally to treat lung injury, obviating the need to administer the MSCs themselves.

However, the current study suggests that the MSC secretome cannot fully replicate the effects of MSC therapy, at least in the early recovery phase following VILI. For example, it is important to point out that MSC-CM had an effect on vascular leak, and at least a partial effect on reducing total inflammatory cell infiltration. These findings provide further indication of the complex mechanism of action of MSCs during injury and repair, rather than evidence against the importance of secreted mediators. In this regard, a number of recent insights into MSC biology should be considered. First, MSCs have important beneficial effects that are cell contact dependent, i.e., effects that require contact between the MSC and another cell. For example, cell-to-cell contact dependent effects may interact with soluble factors to induce MSC-mediated immune suppression [33], and in particular inhibition of T-cell proliferation [34] and macrophage reprogramming to an anti-inflammatory phenotype [1]. In addition, MSCs may enhance repair of the injured alveolar epithelium by direct mitochondrial transfer [13]. Second, MSCs themselves may generate more stable, sustained release of relevant secreted mediators at injury sites in comparison to systemic administration of individual protein factors, which may be subject to immediate metabolism in vivo and may not reach sufficient concentrations at sites of tissue damage. Finally, MSCs are activated by cytokines and chemokines in the injury microenvironment, which can enhance their reparative and immunomodulatory potential [35].

We are not aware of a secreted molecule of importance that could have been removed by the method we have used to produce conditioned medium and concentrate it for in vivo use. It is important to point out that our method for conditioned medium production and concentration is an accepted one [36] and has demonstrated a therapeutic effect in previous studies we have carried out [8, 17] and by other investigators [37]. The centrifugal filters for concentration are characterized by a membrane with a molecular weight cutoff; that is, their ability to retain molecules above a specified molecular weight. Solutes with molecular weights above 3 kDa were retained by our device, which is considerably more sensitive than the 10-kDa filter, as used in many other studies.

#### Limitations

There are a number of limitations to these studies. Firstly, it is difficult to determine whether recovery after injury constitutes solely a repair process or whether cessation of ongoing injurious processes by cell therapy also contributes to the effect seen. Repair after an injurious process such as VILI—reabsorption of edema fluid, alveolar epithelial cell spreading and proliferation, plasma membrane wound sealing—occurs in conjunction with injury [38]. As such there is considerable overlap between injurious and reparative processes. For example, pneumocyte proliferation and collagen synthesis occurs early after institution of mechanical stretch-induced injury and is correlated with survival [9, 39]. However, other than removal of the injurious stimulus, it is difficult to clearly determine which process predominates. We have based the use of the term ‘recovery’ on the fact that the injurious stimulus has been removed (VILI) and MSCs are administered after a demonstrable injury has been established. While damaging inflammation may be ongoing, particularly in the early phase after VILI [9], this inflammation is necessary for an orchestrated repair response [40]. As such, injury and repair are closely linked in this model. Secondly, caution must be exercised in extrapolating from insights in rodent models to the clinical situation. However, these findings add to a growing body of evidence suggesting that MSC therapy may have therapeutic potential for ARDS. Thirdly, we did not provide baseline data on these animals, to allow the reader to assess the magnitude of effects of injurious ventilation on the parameters measured. However, the effect of the high stretch ventilation strategy used in this model has been well characterized in a prior publication from our group [9]. Furthermore, we do not provide data for the effects of MSCs on protectively ventilated or unventilated lungs. However, it would be expected that any effects on these uninjured animals would be limited. Finally, we have not provided a mechanism for how beneficial effects occurred in the MSC-treated group versus the conditioned medium group, which will require further study.

## Conclusions

In conclusion, we have used our established rodent model of recovery after VILI to investigate the effects of rat MSCs on early injury and repair after VILI. We have demonstrated that rodent MSCs, but not the MSC secretome alone, augment lung repair in the early phases after VILI.

### Key message

MSC therapy enhances early recovery following ventilator-induced lung injury in part via a paracrine mechanism.
